# The Navigation Ability Test (NAT 2.0): From Football Player Performance to Balance Rehabilitation in Chronic Unilateral Vestibular Loss

**DOI:** 10.3390/audiolres12030026

**Published:** 2022-05-10

**Authors:** Paolo Gamba, Riccardo Guidetti, Cristiano Balzanelli, Maurizio Bavazzano, Andrea Laborai

**Affiliations:** 1 Department of Otorhinolaryngology, Head and Neck Surgery, LAB of Clinical and Instrumental Vestibology, Poliambulanza Foundation Hospital, 25124 Brescia, Italy; 2 Vertigo Center, Poliambulatorio Chirurgico Modenese, 41125 Modena, Italy; guidetti.r82@gmail.com; 3 Vertigo Center, San Bernardino Polyclinic of Salò, 25087 Brescia, Italy; balzanelli.cristiano@gmail.com; 4 Department of Otorhinolaryngology, Head and Neck Surgery, Clinical and Instrumental Vestibology, San Martino Polyclinic of Genova, 16132 Genova, Italy; mauriziobavazzano39@gmail.com; 5 Department of Otorhinolaryngology, Head and Neck Surgery, Guglielmo da Saliceto Hospital of Piacenza, 29121 Piacenza, Italy; dott.andrealaborai@gmail.com

**Keywords:** spatial working memory, labyrinth memory, topokinetic memory, visual–spatial working memory, Navigation Ability Test (NAT and NAT 2.0)

## Abstract

Aim of the Study: in humans, spatial orientation consists of the ability to move around the environment through memorized and pre-programmed movements, according to the afferent sensory information of the body and environmental analysis of the Central Nervous System (CNS). The purpose of this study is to analyze the abilities of professional athletes, such as footballers, to use mental navigation systems, cognitive maps, and memorized motor patterns in order to obtain better physical performance and to obtain useful information for training both non-sports subjects and vestibular patients for rehabilitation purposes. Materials and Methods: all the motor performances of sportsmen, healthy non-sporting subjects, or vestibular patients are based on the acquisition of visual–spatial and training information. In this study, we analyzed the visual–spatial performance of 60 trained sportsmen (professional footballers), 60 healthy non-sports subjects, and 48 patients affected by chronic unilateral vestibular loss by means of the Navigation Ability Test 2.0. A score based on the number of targets correctly reached in the various tests quantifies the degree of performance of the subjects. Results: NAT 2.0 scores progressively improve from vestibular subjects to healthy non-sporting subjects to footballers. NAT 2.0 scores improve in all three subject groups as the number of tasks performed in all patient groups increases, regardless of gender and age. Conclusions: the analysis of performance data through NAT 2.0 in athletes (footballers) opens new perspectives for rehabilitation purposes, regardless of age, sex, and training conditions, both in healthy non-sporting subjects to improve their sporting potential and in patients affected by chronic vestibular dysfunction, in order to optimize their motor skills and prevent falls.

## 1. Introduction

As we know, a series of reflexes originate from the vestibular system (vestibulo-oculomotor Reflex—VOR; cervico-oculomotor Reflex—COR; vestibulo-spinal Reflex—VSR), allowing the subject to move in the changing surrounding environment, under control of complex cognitive functions, such as memory, attention, visual and mental representation, navigation, etc. [1,2]. This is possible thanks to several neural connections between both labyrinths, vestibular nuclei, cerebellum, hippocampus, prefrontal, and parietal cortex brain areas [3,4]. The ability to quickly process all afferent and efferent somatic sensory information while performing simple or more complex cognitive tasks is called Working Memory (WM) [5,6]. WM represents a refined system, which is able to store the internal and external mental body representation in order to manage a short-term motor program [7,8]. There are different types of WM: long-term and short-term, visual (VWM) and spatial (SWM), which allow the individual to carry out a detailed space, time, and situation analysis in order to achieve a rapid, optimal organization of information. In particular, VWM and SWM are demonstrated to be able to manage the motor skills of adequately trained patients in an age-independent way [9]. They allow the subject to recognize one’s position in space using the hippocampus, in a sort of both subjective and objective topographical representation. In humans, their neuro-anatomical substrate consists of a complex neural network that includes the hippocampus, the anteroposterior area of the parietal lobe, the premotor area, and the prefrontal areas [10]. Patients with chronic vestibulopathy have VWM and SWM deficiency as a direct consequence of labyrinthine deficiency and hippocampal atrophy, which is proportional to the degree of the impairment [11,12]. Experience and training increase the activity of the hippocampus and remove sex-related differences [13,14].

The most widely used tool in the clinical setting for the evaluation of VWM is the Corsi test, which consists of visually recalling a sequence of wooden cubes shown by the examiner, arranged in an irregular pattern on a table: the subject is asked to reproduce the sequence by touching the blocks in the same order. Currently, digital versions of the test eCorsi are available, performed through the use of touch screens. The sequence that corresponds to the maximum number of cubes remembered correctly corresponds to the visual memory span of the subject [15].

The Walked Corsi Test introduces the concept of environmental navigation into the Corsi test. The arrangement of the blocks is displayed on a large surface and the subject, after seeing the sequence of illuminated tiles, has to retrace it, reaching them one after the other by walking, following the visually memorized sequence [16].

An evolution of the Walked Corsi test is the Navigation Ability Test (NAT), which stems out of the concept of training the hippocampus thanks to its relationship with the vestibule. The subject acquires visual and motor information with their eyes open but must retrace the memorized path with their eyes closed, using exclusively hippocampal and labyrinthine information, thus providing data on the SWM [17]. Since damage to the vestibular system leads to cognitive deficits in spatial learning, memory, and navigation, making the mental representation of the surrounding three-dimensional space more difficult, it is well documented how adequate training in spatial orientation with closed eyes can aid recovery of cognitive functions such as visuospatial functions, navigation, and memory [18,19,20,21,22]. NAT is a multimodal and total body stimulation. It is closely connected with the eyes, the control of ocular movements, the head, the movements of the whole body, the posture, and gait. The same functions and information are involved in sports, in particular soccer, which involves smooth pursuit, optokinetic reflex, ocular vergence movements and saccades, reaction speed, and coordination of complex motor movements.

In general, athletes, basketball players, and rugby players develop particular skills depending on their specific training: foot–eye coordination is crucial in football; hand–eye coordination is crucial in basketball and volleyball. Athletes have better stereoscopic visual acuity than non-sports subjects [23,24,25,26]. In order to maximize the athletic-sporting performances, adequate visual training is mandatory, which is also essential in vestibular patients.

## 2. Materials and Methods

**Figure 1:**
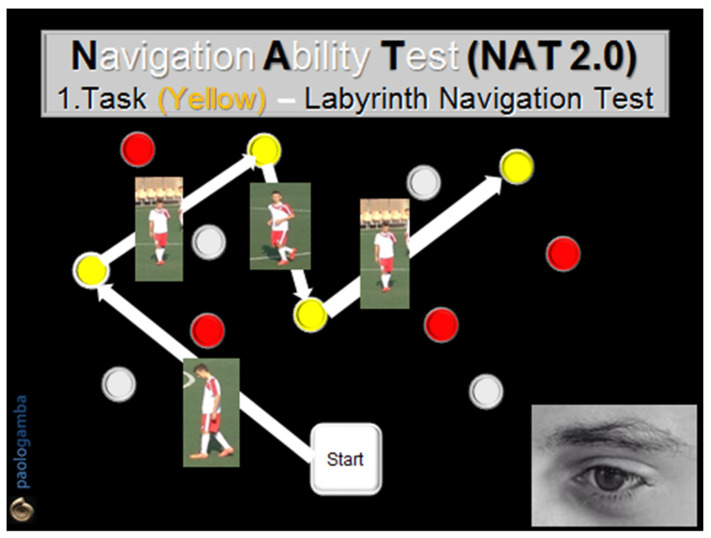
Navigation Ability Test (NAT 2.0). Task 1 (yellow): the subject visually memorizes and then walks with closed eyes along a path drawn by some yellow numbered obstacles located on the ground in front of him/her. The task is performed twice, in both directions, reaching one obstacle after another, according to the numerical order previously visually detected.

**Figure 2:**
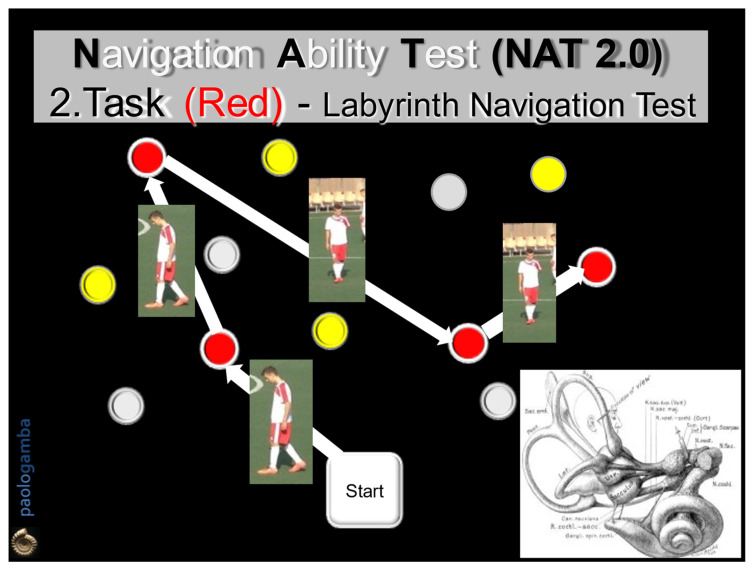
Navigation Ability Test (NAT 2.0). Task 2 (Red): the subject will actively walk with open eyes along a second path, marked by red numbered cones, in the two directions of travel, for 2 consecutive times that must be then repeated with closed eyes. In the absence of visual control, the subject, instant by instant, must draw only on the previously acquired labyrinthine information, expression of the SWM.

The analysis of SWM in sportsmen (football players), healthy non-sports subjects, and vestibular patients is the matter of our study. The main test to investigate navigation and spatial orientation is the Navigation Ability Test (NAT). NAT is an evolution of the Walked Corsi Test, which consists of walking with open eyes along a sequence of previously illuminated and identified blocks, reaching them one by one. It currently has 2 versions, NAT and NAT 2.0. In NAT, the subject walks following a predetermined geometric path (triangle, square, round), first with open eyes, then with closed eyes. In NAT 2.0, the subject undergoes more complex paths (segmented trajectories) with two different tasks (Figure 1 and Figure 2): in both modalities, the subject is invited to observe and acquire visual information looking at the pathway; in the first one, he/she will try to walk with closed eyes on the pathway, but in the second one he/she will have to walk along the path first with open eyes, then with closed eyes, trying to remember all the visual, vestibular, and proprioceptive information previously memorized. The two tests evaluate both the VWM and the SWM, as the visual information elicits the labyrinthine-hippocampal areas and cognition of head and body position, direction, and speed, moment by moment, as a precise “Globe-Positioning-System” Device (GPS-reader).

In our study, we decided to use NAT 2.0. Each subject was invited to observe and memorize two different colored confusing tracks. In the first task, he/she will try to walk along the memorized path; in the second task, he/she will try to walk first with open eyes (yellow-colored task) and then with closed eyes (red-colored task). All tested individuals improved their performance at the second task, suggesting that the best performance is obtained only after both visual and spatial memorization of information. In particular, the performance of the players was significantly better than that of the healthy non-sporting group. In subjects with chronic unilateral vestibular deficit, the visual system alone is unable to create correct spatial maps due to nystagmus or VOR dysfunction, inevitably leading patients to become lost in the track. Their navigation in the dark is precarious and they are forced to activate hippocampal areas and short-term spatial memory [27]. All errors seem to improve with NAT 2.0 training (Table 1).

By means of NAT 2.0, we examined three groups of subjects with completely different age, clinical conditions, cognitive abilities, and motor skills inviting them to walk with closed eyes along a sequence of numbered or colored cubes after visual memorization of the path:

- *Group 1* (*GR 1*): 48 patients affected by chronic unilateral vestibular loss, 24 males and 24 females, average age 71 years (range: 58–87). All patients presented dizziness and chronic imbalance and they were diagnosed by means of a video head impulse test (VHIT) in order to assess the amplitude of the vestibulo-oculomotor reflex (VOR) at a high frequency of impulsive stimulus of the head. Relative VOR gain values of the lateral semicircular canal (LSC) were considered. Inclusion criteria of subjects into GR 1 were: unilateral LSC-VOR amplitude less than 0.8 at VHIT; chronic vestibular hypofunction of more than 6 months. Exclusion criteria were: Previous otosurgery, Benign Paroxysmal Positional Vertigo (BPPV), Meniere Disease, neurological diseases, and chronic use of Benzodiazepines and/or Neuroleptics.
- *Group 2* (*GR 2*): 60 healthy patients, not sportsmen, 30 males and 30 females, average age 48 ears (range 24–58). They were all asymptomatic and presented normal LSC-VOR at VHIT.
- *Group 3* (*GR 3*): 60 professional football players (Brescia Football men Division B, Brescia Football Women’s Division B and Cremonese Football men Division under 21), 32 males and 28 females average age of 25 years, (range 16–31). They were all asymptomatic and presented normal LSC-VOR at VHIT.

All subjects were invited to observe, memorize, and walk along two different colored paths marked by colored obstacles, in both directions, first with open eyes and then with closed eyes, following the NAT 2.0 procedure. The number of correctly reached obstacles in both directions of the run, with open and closed eyes, was considered. Informed consent before the test was obtained by each participant. In order to evaluate the cognitive abilities related to SWM, NAT was performed in two modalities:

- The 1st task (Labyrinth Navigation Task—LNT, yellow-colored path): in a two-step modality, at a distance of 1 m, the subject was first invited to memorize a yellow-colored path on the ground and then to walk along it with closed eyes 2 consecutive times, in both directions, reaching one obstacle after another, according to the numerical order previously visually detected (Figure 1).
- The 2nd task (Labyrinth Navigation Task—LNT, Red-colored path): in a three-step modality, the subject was invited to memorize a second red-colored path on the ground, then to walk along it first with open eyes in both directions 2 consecutive times and then with closed eyes. In the closed-eyes condition, moment by moment, the subject reconstructs the previously memorized map, exploiting only the labyrinthine information—an expression of the SWM (Figure 2).

We considered the number of obstacles correctly reached in both directions of walking, both in test and re-test. The performance results were collected for all three groups of subjects according to score, ranging from 0 to 4, based on the number of cones correctly achieved: the increase in the score indicates a better execution of the task, in terms of memorization, spatial orientation, and vestibular balance. Scores were collected by personnel with oto-neurological experience (Table 1).

## 3. Results

The NAT 2.0 scores in the subjects of GR1, GR2, and GR3 are shown in Table 1, and they are summarized as follows:

- GR1 (Dizzy subjects): scores 2 and 2.3, respectively, for the LNT yellow-colored path test and re-test; scores 1.5 and 2.1, respectively, for the LNT red-colored path test and re-test;
- GR2 (Healthy non-sports subjects): scores 3.1 and 3, respectively, for the LNT yellow-colored path test and re-test; scores 2.4 and 2.6, respectively, for the LNT red-colored path test and re-test;
- GR3 (Football player subjects): scores 3.1 and 3, respectively, for the LNT yellow-colored test and re-test; scores 2.6 and 3, respectively, for the LNT red-colored test and re-test.

Important differences in the scores of GR1 patients compared with the scores of GR2 subjects and GR3 players are shown in Table 1. In particular, LNT yellow and red-colored path scores in GR3 are better than those of GR1 and GR2.

**Figure 3:**
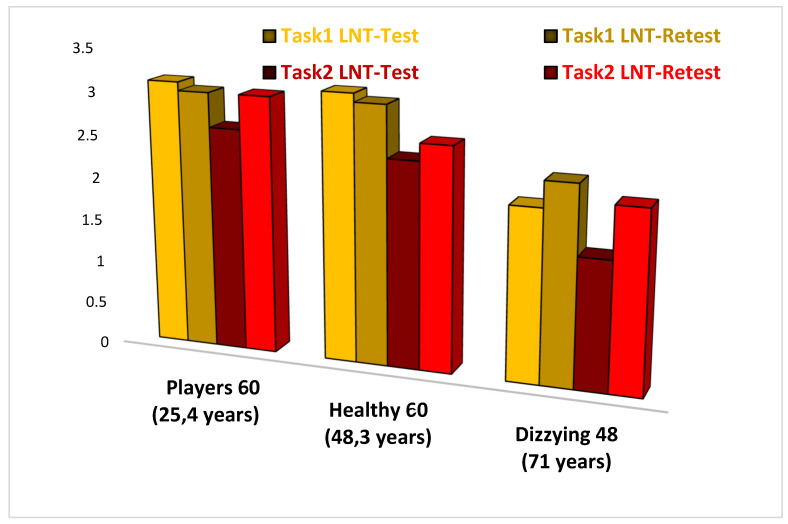
Comparison of NAT 2.0 results in players, healthy non-sporting subjects, and dizzy patients. As it can be seen from the table, there are important differences in the scores of dizzy subjects compared to healthy non-sporting subjects and football players; the latter present Labyrinth Navigation Test scores (LNT), which are better than the other two groups of subjects.

Furthermore, we found:

- Progressively better scores from GR1 to GR3 for both Task 1 (LNT yellow-colored path) and Task 2 (LNT red-colored path), suggesting that both visual and cognitive rehabilitation is essential in vestibular training;
- Test and re-test scores of Task 1 (LNT yellow-colored path) are increased only in GR3, suggesting fast and better results in trained persons;
- Test and re-test scores of Task 2 (LNT red-colored path) are increased in all three groups of subjects, suggesting that pre-programmed and memorized visual information allows for improvement of motor skills in any clinical or training condition;
- Age (significantly higher range in GR1 than in GR2 and GR3) did not affect the performance results in the LNT-Tasks in the three groups of examined subjects: these data suggest that NAT rehabilitation allows safer and effective motor performances, improving balance control in subjects of any age and in any physical training condition (Figure 3).

## 4. Discussion

Our study confirms that at any age, both in sports and in pathology, navigation with closed eyes improves short-term spatial memory and makes the labyrinthine memory equally effective as visual memory. Our hypothesis is that an improvement of SWM in a vestibular rehabilitative setting may induce functional changes in the hippocampus and parietal brain cortex, possibly through direct pathways with the vestibular system. Patients with chronic vertigo may benefit from a combination of visual and motor rehabilitation and from the use of nootropic brain stimulation drugs [28]. The different ability of the CNS to adapt to new and/or dangerous situations in the case of vestibular dysfunction depends on each person’s own individual neuroplastic reprogramming of SWM. Vestibular compensation is not constant over time and depends on many individual factors, which must always be taken into consideration (age, sex, emotions, stress, drugs, habits, comorbidity). For this reason, the vestibular patient should not be confined to bed, but it is mandatory to facilitate an early start of dynamic experiences as is possible, reducing the risk of fear and traumatic memory [29,30,31].

**Figure 4:**
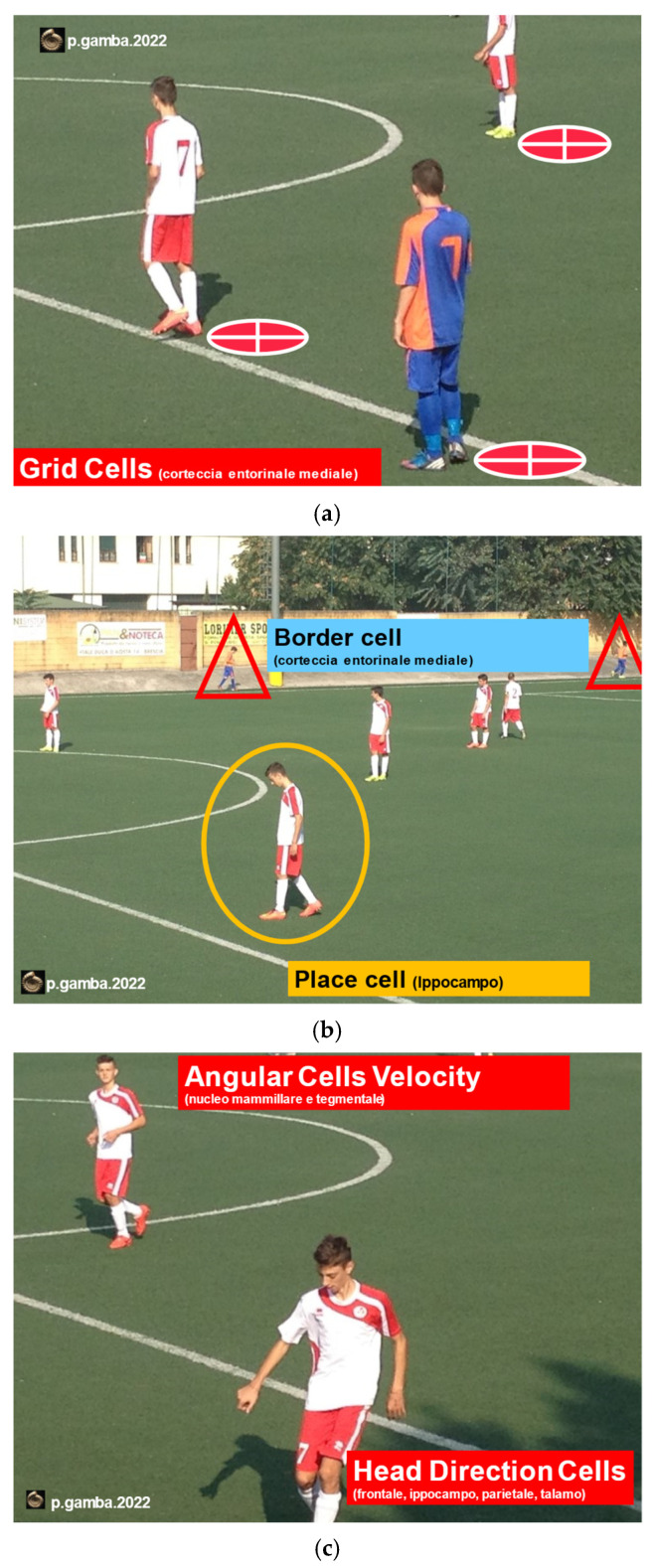
CNS cells specialized in spatial orientation: *Grid Cells* and the *Border Cells*, located in the medial entorhinal cortex (**a**); *Place Cells*, located in the hippocampus (**b**); *Head Direction Cells* (HD), located in the frontal cortex, in the hippocampus, parietal cortex, and thalamus, and indicate the direction of the head; the *Angular Velocity Cells* (AVC), located in the mammillary and tegmental nucleus and indicate the speed of rotation of the head (**c**).

Our data support the concept that during one’s movement in space, both in healthy and/or sports subjects, the CNS updates the self-position by acquiring plenty of visual reference points, in combination with proprioceptive feedback and memorized pre-programmed motor control [32,33,34,35,36,37]. In the vestibular patient, when external landmarks are negated, CNS integration processes could lead to misinterpretation of both direction and position between body and objects, causing loss of balance. In fact, in the CNS, four types of cells allow interpretation and decoding of the visual and spatial information at any age and in any condition of physical training: the “*Grid Cells*”, the “*Border Cells*”, the “*Head-Directions Cells*”, and the “*Angular-Velocity Cells*”. Improving their functional integration through NAT and NAT 2.0 training means developing better cognitive control of the situational setting, better spatial orientation, easier navigation, better motor control, and better balance. In a football match, as well as in every daily life activity, the mentioned cells interact actively instant by instant in order to decode and memorize all environmental information, static and dynamic properties of objects in the visual field, and body motion perception (Figure 4). Cognitive deficits, such as poor concentration or short-term memory loss due to neurological dysfunctions, are frequent among patients with vestibular disorders, especially with old age, causing disorientation, imbalance, and falls [38]. On the contrary, damage to the vestibular system inevitably leads to cognitive deficits in learning, visual and spatial memory, with consequent VWM and SWM deficits, altered self-perception of position in space, reduced navigation, and loss of balance [39]. By means of the NAT 2.0 program, the possibility to obtain and quantify the visuo-spatial abilities (VWM and SWM) based on a series of sports and trained subjects, compared to those of healthy non-sports subjects, becomes extremely important for planning vestibular rehabilitation [40].

The SWM is the matter of our study, and it represents an active cognitive process both responsible for recording spatial and environmental information surrounding the subject and his/her body orientation, thanks to the hippocampus, parietal, and prefrontal cortex brain areas activation. The goal is to use environmental references as “landmarks”, to train memory, increase speed in locating visually perceived objects, and change strategy at the right time [41]. The SWM allows one to remember places, distances, and spatial relationships among objects and to elaborate at any moment on the most suitable way to reach them. In fact, everyone can easily move in familiar and well-known settings due to memory of the surrounding environment, in a manner similar to a detailed map [42].

In all subjects, visual–spatial perception allows interaction with the surrounding environment. The vestibular system, i.e., proprioceptive, tactile, and auditory systems, participates in the complex CNS process of instant-by-instant integration, elaboration, comparison, and memorization of any visual and spatial information. If space information is perceived as altered, as in vestibular dysfunction, posture and balance control could also be wrong [43,44]. Visuo-spatial training by means of NAT and NAT 2.0 represents an important task to develop the self-perception of the body in space, both to improve motor skills in sportsmen and to restore a healthy situation in vestibular patients. The NAT 2.0 experience of athletes and our data analysis of their performances open new perspectives for rehabilitative therapy of vestibular patients, who need to improve the same processes of VSM and SWM: in fact, in order to improve their motor abilities, both the players and the vestibular patients need to memorize and develop predetermined paths (triangles, squares, circles: NAT) or more complex paths (segmented trajectories: NAT 2.0), repeated in a number of series with open and closed eyes. The aim of sports training coincides with those of visuo-vestibular rehabilitation: learning, commitment (memory), and attention (arousal) [45,46].

The limitation of using NAT as a tool to assess the subjects’ visual–spatial capabilities is that the data processing is not software-based. This study allows the basis for future research in order to record and compare the rehabilitation results of the various forms of primary or secondary vestibular dysfunction.

## 5. Conclusions

NAT 2.0 scores are progressively better in vestibular patients (GR1), healthy non-sports subjects (GR2), and football players subjects (GR3) in Task 2 (re-test) than in Task 1 (1st test), and in the open-eyes condition compared to closed eyes, without any age-dependent difference.

These data suggest that SWM evaluation and rehabilitation by NAT 2.0 allow better cognitive control of the situational setting, better spatial orientation, easier navigation, and better motor and balance control, both in sports and vestibular subjects.

NAT 2.0 allows a numerical quantification of the improvement of cognitive and motor performance in all groups of pre and post-rehabilitated subjects, which in future research could be implemented with dedicated software.

We conclude that, due to the close connections of the vestibular system with the CNS, the SWM can be considered an excellent indicator of the degree of cognitive deterioration of the vestibular patient; it also opens new rehabilitation perspectives. In fact, NAT 2.0 rehabilitation could be used to test and train vestibular function, both in the outcome of sports performance in healthy subjects and in chronic vestibular patients, in order to improve their motor skills based on their activities.

## Figures and Tables

**Table 1:** NAT 2.0 scores (0 to 4) for tests and re-tests in dizzy patients (GR1), healthy non-sporting patients (GR2), and football players (GR3); LNT: Labyrinth Navigation Task.

Tasks	GR1 (*Dizzy*) (n = 48)	GR2 (*Heathy*) (n = 60)	GR3 (*Players*) (n = 60)
Task 1: LNT			
*LNT-1 (test)*	*2*	*3.1*	*3.1*
*LNT-2 (re-test)*	*2.3*	*3*	*3*
Task 2: LNT			
*LNT-1 (test)*	*1.5*	*2.4*	*2.6*
*LNT-2 (re-test)*	*2.1*	*2.6*	*3*

## Data Availability

The data presented in this study are available on request from the corresponding authors.
